# Supplementary material for: Identification of photoperiod-induced specific miRNAs in the adrenal glands of Sunite sheep (Ovis aries)
Source: Front Vet Sci. 2022 Jul 22;9:888207. doi: 10.3389/fvets.2022.888207 (PMC9354845; doi:10.3389/fvets.2022.888207)
Supplement: Supplementary file 1 [file Data_Sheet_1.ZIP › Supplementary file/miRDeep2 file.zip!/miRDeep2 file/Novel_Structure/SPLP42A3/Novel_548.pdf]

Provisional ID	:	NC_019469.2_22909
Score total	:	11.1
Score for star read(s)	:	3.9
Score for read counts	:	3.7
Score for mfe	:	1.9
Score for randfold	:	1.6
Score for cons. seed	:	
Total read count	:	19
Mature read count	:	12
Loop read count	:	0
Star read count	:	7

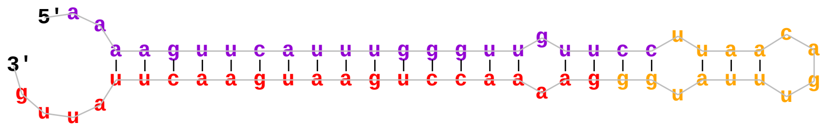

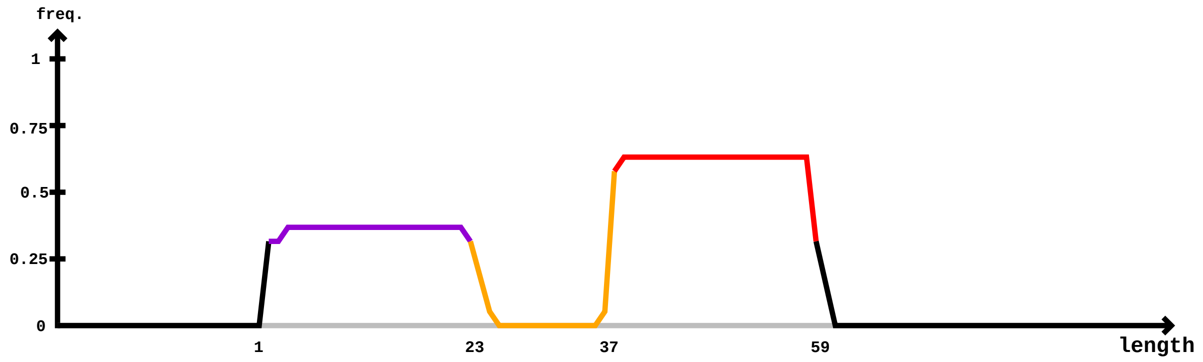

Star **Mature**

```
5'- uacuacuauuguguuggccaaaaaguucauuuggguuguuccuuaacaguuuaugggaaaaccugaaugaacuuauuggucaacccaauacgucagagaaauauagagugacag   -3'   obs
    uacuacuauuguguuggccaaaaaguucauuuggguuguuccuuaacaguuuaugggaaaaccugaaugaacuuauuggucaacccaauacgucagagaaauauagagugacag         exp
    ......(((((.(((((((((.(((((((((((((((.((((.(((....))).)))).))))))))))))))).))))))))).))))).((((.............))))..   reads   mm   sample
    ....................aaaaguucauuuggguuguuc.........................................................................   1       0    seq
    ....................aaaaguucauuuggguuguucc........................................................................   3       0    seq
    ....................aaaaguucauuuggguuguuccA.......................................................................   1       1    seq
    ....................aaaaguucauuuggguuguuccuu......................................................................   1       0    seq
    ......................aaguucauuuggguuUuucc........................................................................   1       1    seq
    .......................................................ggaaaaccugaaugaacuuUuu.....................................   1       1    seq
    ........................................................gaaaaccugaaugaacuuauu.....................................   3       0    seq
    ........................................................gaaaaccugaaugaacuuUuu.....................................   1       1    seq
    ........................................................gaaaaccugaaugaacuuauug....................................   6       0    seq
    .........................................................aaaaccugaaugaacuuauu.....................................   1       0    seq
```
